# PPARβ/δ Is Required for Mesenchymal Stem Cell Cardioprotective Effects Independently of Their Anti-inflammatory Properties in Myocardial Ischemia-Reperfusion Injury

**DOI:** 10.3389/fcvm.2021.681002

**Published:** 2021-09-20

**Authors:** Nitirut Nernpermpisooth, Charlotte Sarre, Christian Barrere, Rafaël Contreras, Patricia Luz-Crawford, Gautier Tejedor, Anne Vincent, Christophe Piot, Sarawut Kumphune, Joel Nargeot, Christian Jorgensen, Stéphanie Barrère-Lemaire, Farida Djouad

**Affiliations:** ^1^Institut de Génomique Fonctionnelle, Université de Montpellier, CNRS, INSERM, Montpellier, France; ^2^Department of Cardio-Thoracic Technology, Faculty of Allied Health Sciences, Integrative Biomedical Research Unit, Naresuan University, Phitsanulok, Thailand; ^3^Institute for Regenerative Medicine and Biotherapy, Université de Montpellier, INSERM, Montpellier, France; ^4^Laboratorio de Inmunología Celular y Molecular, Facultad de Medicina, Universidad de los Andes, Santiago, Chile; ^5^MedXCell Science, Institute for Regenerative Medicine and Biotherapy, Montpellier, France; ^6^Département de Cardiologie Interventionnelle, Clinique du Millénaire, Montpellier, France; ^7^Biomedical Engineering Institute, Chiang Mai University, Chiang Mai, Thailand; ^8^Centre Hospitalier Universitaire Montpellier, Montpellier, France

**Keywords:** myocardial infarction, reperfusion injury, inflammation, PPAR, mesenchymal stem cells, cardioprotection

## Abstract

Myocardial infarction ranks first for the mortality worldwide. Because the adult heart is unable to regenerate, fibrosis develops to compensate for the loss of contractile tissue after infarction, leading to cardiac remodeling and heart failure. Adult mesenchymal stem cells (MSC) regenerative properties, as well as their safety and efficacy, have been demonstrated in preclinical models. However, in clinical trials, their beneficial effects are controversial. In an experimental model of arthritis, we have previously shown that PPARβ**/**δ deficiency enhanced the therapeutic effect of MSC. The aim of the present study was to compare the therapeutic effects of wild-type MSC (MSC) and MSC deficient for PPARβ**/**δ (KO MSC) perfused in an *ex vivo* mouse model of ischemia-reperfusion (IR) injury. For this purpose, hearts from C57BL/6J mice were subjected *ex vivo* to 30 min ischemia followed by 1-h reperfusion. MSC and KO MSC were injected into the Langendorff system during reperfusion. After 1 h of reperfusion, the TTC method was used to assess infarct size. Coronary effluents collected in basal condition (before ischemia) and after ischemia at 1 h of reperfusion were analyzed for their cytokine profiles. The dose-response curve for the cardioprotection was established *ex vivo* using different doses of MSC (3.10^5^, 6.10^5^, and 24.10^5^ cells**/**heart) and the dose of 6.10^5^ MSC was found to be the optimal concentration. We showed that the cardioprotective effect of MSC was PPARβ**/**δ-dependent since it was lost using KO MSC. Moreover, cytokine profiling of the coronary effluents collected in the eluates after 60 min of reperfusion revealed that MSC treatment decreases CXCL1 chemokine and interleukin-6 release compared with untreated hearts. This anti-inflammatory effect of MSC was also observed when hearts were treated with PPARβ**/**δ-deficient MSC. In conclusion, our study revealed that the acute cardioprotective properties of MSC in an *ex vivo* model of IR injury, assessed by a decreased infarct size at 1 h of reperfusion, are PPARβ**/**δ-dependent but not related to their anti-inflammatory effects.

## Introduction

Acute myocardial infarction (AMI) is the leading cause of cardiovascular mortality worldwide and a provider of heart failure (1). Prompt revascularization of the culprit artery with primary coronary angioplasty or thrombolysis is associated with deleterious side effects called ischemia-reperfusion (IR) injury due to abrupt restoration of blood flow and oxygen.

The release of DAMPS (*Damage-associated molecular patterns*) from dead cells, in concert with the activation of the complement cascade and reactive oxygen species (ROS), triggers an acute pro-inflammatory response at the onset of AMI that activates the resident immune cells of the heart. Reperfusion exacerbates this inflammatory response to eliminate necrotic cells and repair the infarcted myocardium (2). Interleukin 1 (IL-1), and interleukin 6 (IL-6) are the major cytokines that mediate this short but strong inflammatory burst, which contributes to cell death and irreversible IR injury (2). At the site of the injury, many cell types, including cardiomyocytes, vascular cells, fibroblasts, and immune cells, are involved in this inflammatory response. Necrotic cardiomyocytes in the infarcted area provide the main stimulus for the post-infarction inflammatory response through the release of DAMPs. In the border zone, surviving cardiomyocytes, once activated by IL-1, Toll Like Receptor (TLR) ligands, and ROS, will produce and secrete cytokines such as IL-6 (3), TNFα (4), and chemokines such as CXCL1 (KC**/**GRO) and MIP-2 (5) to trigger inflammatory activation. Endothelial cells, the most abundant non-cardiomyocytes in the heart, when activated by TNFα, produce CXCL1 (6). In addition to a local inflammatory response, myocardial cells sense tissue necrosis and trigger the post-infarction inflammatory response, stimulating the release and recruitment of bone marrow (BM)-derived leukocytes. However, the relative contribution of cardiomyocyte-derived inflammatory mediators in the progression and extension of post-infarction inflammation remains unknown.

Previous studies have shown that the application of ischemic postconditioning (PostC) i.e., repeated brief episodes of IR in the myocardial tissue applied at the onset of reperfusion, was able to specifically inhibit IR injury (7, 8). PostC, considered a gold standard strategy for cardioprotection in animal models of AMI, was reported to be mediated by multiple intracellular cascades leading to anti-apoptotic and anti-inflammatory effects resulting in cardioprotection (7, 9, 10). Various targets have been identified in PostC signaling pathways, however, no product of potential clinical utility, including anti-inflammatory drugs, has emerged from all candidates identified as cardioprotective in preclinical studies (11, 12). This suggests that other strategies with pleiotropic mechanisms of action are clearly needed (13).

Preclinical studies have shown that MSC-based therapy improves myocardial functional recovery after AMI by promoting endogenous cell survival, proliferation, and angiogenesis. In addition, MSC exert pleiotropic effects, including reduction of inflammation and apoptosis through their ability to release bioactive molecules (14, 15). Based on these promising results, MSC were then tested in clinical trials that demonstrated their safety and promising efficacy in phase I and II, but yielded inconclusive results in phase III trials (16). Indeed, no significant long-term beneficial effects in AMI patients has been reported based on recent meta-analyses (17, 18).This failure to translate preclinical results into human clinical trials could be attributed to, in part, trial design differences, the source and dose of MSC used, and the route and timing of MSC injection (18). To bridge the gap between preclinical and clinical studies, the development of “preconditioning” methods to improve MSC therapeutic potential has been widely investigated (19) mainly focusing on the enhancement of their anti-inflammatory properties. For example, MSC treated with IGF-1 before transplantation into the ischemic heart reduce the production and expression of proinflammatory cytokines, including TNFα, IL-1β, and IL-6 and improve cardiac functions (20). Although promising, this approach of enhancing the anti-inflammatory properties of MSC to improve their therapeutic potential in AMI has been poorly investigated.

Peroxisome proliferator-activated receptors (PPARs) are nuclear receptors expressed in three different isoforms, PPARα, PPARβ**/**δ, and PPARγ, which heterodimerize with the retinoid X receptor (RXR) and act as transcriptional regulators after ligand binding. Peroxisome proliferator-activated receptor isoforms exert multiple functions depending on tissue ligands and cofactors (21). PPARβ**/**δ, a proangiogenic member of the PPAR family, is ubiquitously expressed (22–24) in contrast to PPARα, which is mainly detected in brown adipose tissue, intestine, heart, liver, kidney, and PPARγ, which is expressed in immune cells, intestine, white, and brown adipose tissue. The potent anti-inflammatory actions of PPARβ**/**δ on several immune cells including macrophages have been previously reported. Indeed, the capacity of IL-4 and IL-13 to direct macrophages to an M2-like anti-inflammatory phenotype in mouse adipose tissue and liver depends on PPARβ**/**δ expression (25–27). Recently, in an experimental model of the auto-immune and inflammatory disorder in mice, “collagen-induced arthritis (CIA),” we demonstrated that PPARβ**/**δ expression level could predict the immunoregulatory potential of MSC and that its inhibition increased their immunoregulatory and therapeutic activities (28).

Given that reperfusion injury is associated with acute inflammation, we hypothesized that inactivation of PPARβ**/**δ might impact the cardioprotective properties of MSC during IR injury associated to local inflammation (26). In mouse models, in order to get closer to the classical clinical conditions of MSC administration (29–37), the local injection of MSC at the acute phase, avoiding the systemic route is prefered. Thus, in the present study, we explored the contribution of PPARβ**/**δ in the acute local cardioprotective effect mediated by MSC during reperfusion in an *ex vivo* mouse model of isolated heart subjected to IR injury.

## Materials and Methods

### Ethics

Studies involving animals were reviewed and approved by the Institute's SBEA (*Structure Bien-être Animal*) commitee in accordance with the European directive 2010**/**63**/**EU and the French Ministerial Order of February 01, 2013.

### Animal Housing and Care

Experiments were performed in C57BL**/**6J mice (Charles River laboratory) in accordance with the European Communities Council directive of November 1986 and in accordance with the Guide for the Care and Use of Laboratory Animals" published by the US National Institutes of Health (NIH publication 8^th^ Edition, 2011). All mice were maintained under controlled environmental conditions (22 ± 2°C, 12 h light **/**12 h dark cycle) in the Institute's animal facility.

### *Ex vivo* Experiments

Male mice were anesthetized with an intraperitoneal injection (IP) of ketamine (14 mg/kg, Imalgène® *Merial*), xylazine (14 mg/kg, Rompun® *Bayer*) followed by an injection of pentobarbital (IP; 76.6 mg/kg; *Sanofi-Aventis*). The anesthetized mice received 250 U heparin (IP) in order to prevent blood clot formation. After sternotomy, the heart was excised, cannulated through the ascending aorta, and quickly mounted on the Langendorff perfusion system. Prewarmed Tyrode's solution (NaCl 140 mM, KCl 5.4 mM, MgCl 1 mM, Hepes 5 mM, glucose 5.5 mM, CaCl_2_ 1.8 mM, pH 7.4) was perfused at constant pressure (70 mmHg) and temperature (37°C).

### Ischemia-Reperfusion Protocol

On the Langendorff perfusion system, the heart was perfused with prewarmed Tyrode solution for 15 min (stabilization). Global ischemia was obtained by stopping the perfusion flow (*no-flow*) for 30 min. A reperfusion step (60 min) was achieved by restoring the flow. Mesenchymal stem cells treatment (prepared in Tyrode solution) was applied during reperfusion as a non-recirculating perfusate. The control condition (IR) was obtained using only Tyrode. A positive control of cardioprotection was obtained by applying an ischemic postconditioning stimulus, comprising three cycles of 1 min ischemia-1 min reperfusion at the onset of reperfusion (PostC group). Perfusates containing coronary effluents were collected at the apex of the heart at both 10 min of the stabilization (basal) phase and at 15, 30, and 60 min after the onset of reperfusion and were stored at −80°C for further experiments.

### Infarct Size Measurement

At the end of the IR protocol, the heart was harvested from the apparatus. The left ventricle (LV) was embedded in agar (4% w/v), and transversely sliced (1 mm) with a vibratome. To reveal tissue viability, slices were incubated in a 1% solution of 2,3,5-triphenyltetrazolium chloride (TTC, *Sigma-Aldrich*) for 15 min at 37°C. After a fixation step (4% paraformaldehyde, 48 h), each slice was photographed from each side. The infarct area was quantified by planimetric measurements with *ImageJ* software.

### MSC Culture

Isolation, amplification, and characterization of murine MSC were performed as previously described (33). Briefly, BM was flushed out from the long bones of *Ppard*^fl/fl^sox2cre^tg^ PPARβ/δ-deficient mice and their wild-type littermates (*Ppard*^fl/+^) kindly provided by Gerhard Krönke laboratory (Institute of Rheumatology and Immunology, Erlangen, Germany) (38) to isolate KO MSC and MSC, respectively. Cells were cultured in minimal essential medium (MEM)-α containing 10% fetal bovine serum, 2 mM glutamine, 100 U/ml penicillin, 100 mg/ml streptomycin, and 2 ng/ml human basic fibroblast growth factor (bFGF) at a density of 0.5 × 10^6^ cells/cm^2^. Phenotypic and functional characterization of MSC has been performed previously (28). To confirm the effects of PPARb/d inactivation, mesenchymal stem cells were pre-incubated 24 h with 5 μM of PPARβ/δ selective antagonist GSK0660.

### MSC Labeling With CM-DiI

The stock solution of the fluorescent cell-tracer CM-DiI (Molecular Probes) was reconstituted in dimethyl sulfoxide (DMSO) at a concentration of 1 μg/μl. Mesenchymal stem cells were collected and suspended at the concentration of 1 × 10^7^ cells/10 μg CM-DiI in 5 ml PBS. Cells were incubated at 37°C for 5 min followed by 15 min at 4°C, in the dark. Unincorporated fluorescent dye was then removed by centrifugation at 300 g for 5 min and two washes with PBS were performed. Labeled cells were resuspended in Tyrode's solution and maintained at 4°C prior being injected into the myocardium.

### Cytokine Level Quantification

For quantification of cytokine levels, coronary effluents from perfused hearts were collected under basal conditions (*t*0), after 10 min of stabilization, and after IR injury at 15, 30, and 60 min after the onset of reperfusion. The different perfusates were stored at −80°C until the assay was performed using the Meso Scale Discovery (MSD) V-Plex Plus Proinflammatory Panel 1 (mouse) kit at the “Plateforme de Protéomique Clinique de Montpellier” according to the manufacturer's protocol. As compared to other methods, MSD is the most suitable assay for samples with low endogenous levels of the cytokines although cytokine's levels can be below the limits of detection of this immunoassays. The V-Plex Plus Proinflammatory Panel 1 includes IFNγ, IL-1β, IL-2, IL-4, IL-5, IL-6, KC/GRO (CXCL1), IL-10, IL-12p70, and TNFα. When the concentration of a cytokine was undistinguishable from background (i.e., below the limit of detection), the sample was considered negative for that cytokine.

### Immunochemistry

At the end of *ex vivo* experiments, LV were fixated in 4%-PFA and embedded in paraffin. Each LV was cut from apex to base (sections of 4 μM each 150 μM). The paraffin-embedded sections were deparaffinized then rehydrated through an alcohol gradient. Left ventricle sections were incubated with a primary anti α-actinin antibody (1:100, mouse monoclonal; *Sigma-Aldrich*). Cell nuclei were stained with Hoechst (*Life technologies SAS*) and endothelial cells with Isolectin B4 (FITC Conjugate; *Sigma-Aldrich*). After incubation with primary antibodies, sections were washed in PBS, and then incubated (3 h) with secondary antibodies (1:2,000, *Jackson ImmunoRes Laboratories*, Inc.). Primary and secondary antibodies were diluted in PBS containing 3% BSA and 0.1% Triton X100. Stained sections were mounted in Mowiol (*Biovalley*). Images were obtained with a Zeiss Axioimager Z3 fluorescent microscope after observation of six different sections of the LV harvested on *n* = 2 hearts treated by MSC labeled with CM-DiI and analyzed using ImageJ and Adobe Photoshop to prepare the final figures.

### Statistical Analysis

Data expressed as mean ± SD values were compared among groups using non-parametric Mann-Whitney (two groups) and Kruskal-Wallis (multiple comparison) methods. *P*-values < 0.05 (^*^), *p* < 0.01 (^**^), *p* < 0.001 (^***^), and *p* < 0.0001(^****^) were considered statistically significant. Analysis and graphical representation were performed using Graph-Pad Prism^™^ software (GraphPad).

## Results

### Induction of a Pro-inflammatory Response in Isolated Perfused Heart Subjected to Ischemia-Reperfusion Injury *Ex vivo*

**Figure 1:**
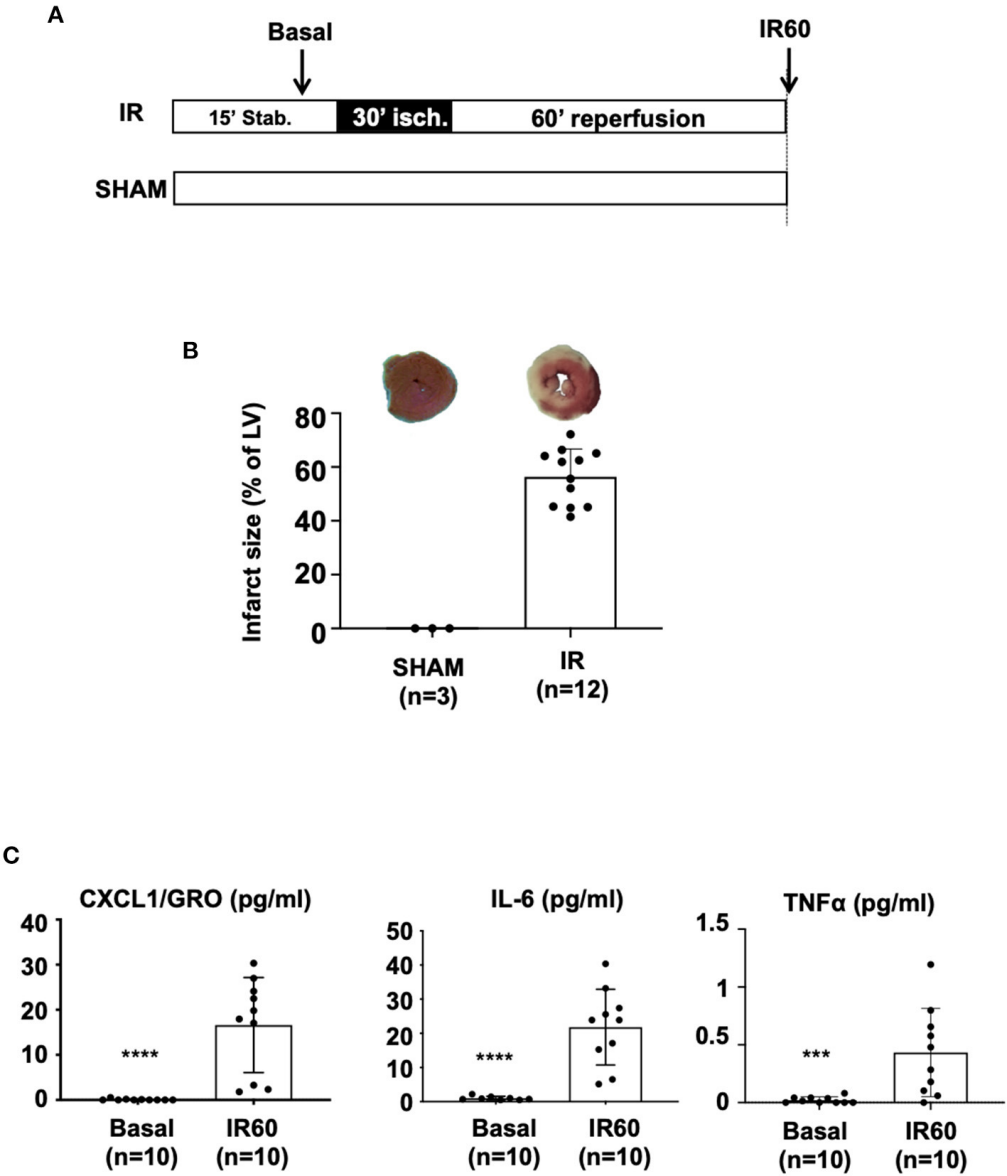
C57BL/6J mouse hearts were mounted on a Langendorff system and subjected to IR injury. **(A)** The *ex vivo* protocol included a 15 min-stabilization period, followed by 30 min of global ischemia achieved by stopping the flow in the aorta (no-flow). Reperfusion was achieved by restoring the Tyrode infusion during 60 min. For the SHAM condition, the heart was perfused throughout the protocol without any ischemic induction. At the end of the protocol, infarct size was measured using the TTC-staining method. Coronary effluents were collected at two time points during the *ex vivo* protocol: during stabilization to evaluate the “basal” level of cytokine release and at the end of the reperfusion phase to evaluate the “IR60” cytokine production after IR injury. **(B)** Scatter plots and bars (mean ± SD) were represented for infarct size (in % of LV) in IR (*n* = 12) and SHAM (*n* = 3) hearts. Representative pictures of TTC-stained LV slices were shown for each group. **(C)** Scatter plots with bars (mean ± SD) are presented for quantification of cytokines within coronary effluents collected before ischemia (Basal) and after 60 min of reperfusion after the IR protocol (IR_60_) using the Meso Scale Discovery (MSD) V-Plex Plus Proinflammatory Panel 1 (mouse) kit. Statistical analysis was performed using the Mann-Whitney test. For CXCL1 (pg/ml), ^****^ was noted for p < 0.0001, for IL-6 (pg/ml), ^****^ was noted for *p*< 0.0001 and for TNFα (pg/ml), ^***^ was noted for *p* = 0.0008.

For this study, we have developed an *ex vivo* model of global ischemia followed by reperfusion (IR protocol) to evaluate the short-term therapeutic effects of MSC. Isolated hearts were mounted and perfused on a Langendorff system and subjected to 30 min of global ischemia (no-flow) followed by 60 min of reperfusion (see protocol in Figure 1A). The first part of the study was devoted to the characterization of our model. Hearts after myocardial IR injury were characterized by an infarct size with a mean value of 56.4% ± 10.3 expressed as percentage of the LV (Figure 1B).

We therefore asked whether induction of IR injury was associated with excessive release of pro-inflammatory cytokines in coronary effluents collected 60 min after the onset of reperfusion (time point at which infarct size was evaluated) compared with the basal level before ischemia (collected during stabilization). Of the 10 cytokines quantified using the MSD VPlex Plus Proinflammatory, seven including IFNγ, IL-1β, IL-2, IL-4, IL-5, IL-10, IL-12p70 were not considered because of their undetectable levels. However, the concentration of three cytokines, KC/GRO (CXCL1), IL-6, and TNFα, were showed to be significantly increased in the coronary effluents of the hearts after IR injury (after ischemia and 60 min of reperfusion) compared with the basal conditions (collected at 10 min stabilization, basal) (Figure 1C). Overall, these results reveal that myocardial IR injury of C57BL/6 mouse hearts subjected *ex vivo* to 30 min of ischemia followed by 1 h of reperfusion is associated with the release of pro-inflammatory cytokines.

### MSC Exerted a Potent Cardioprotective Effect When Administered During Reperfusion in an *Ex vivo* Model of Global Ischemia

**Figure 2:**
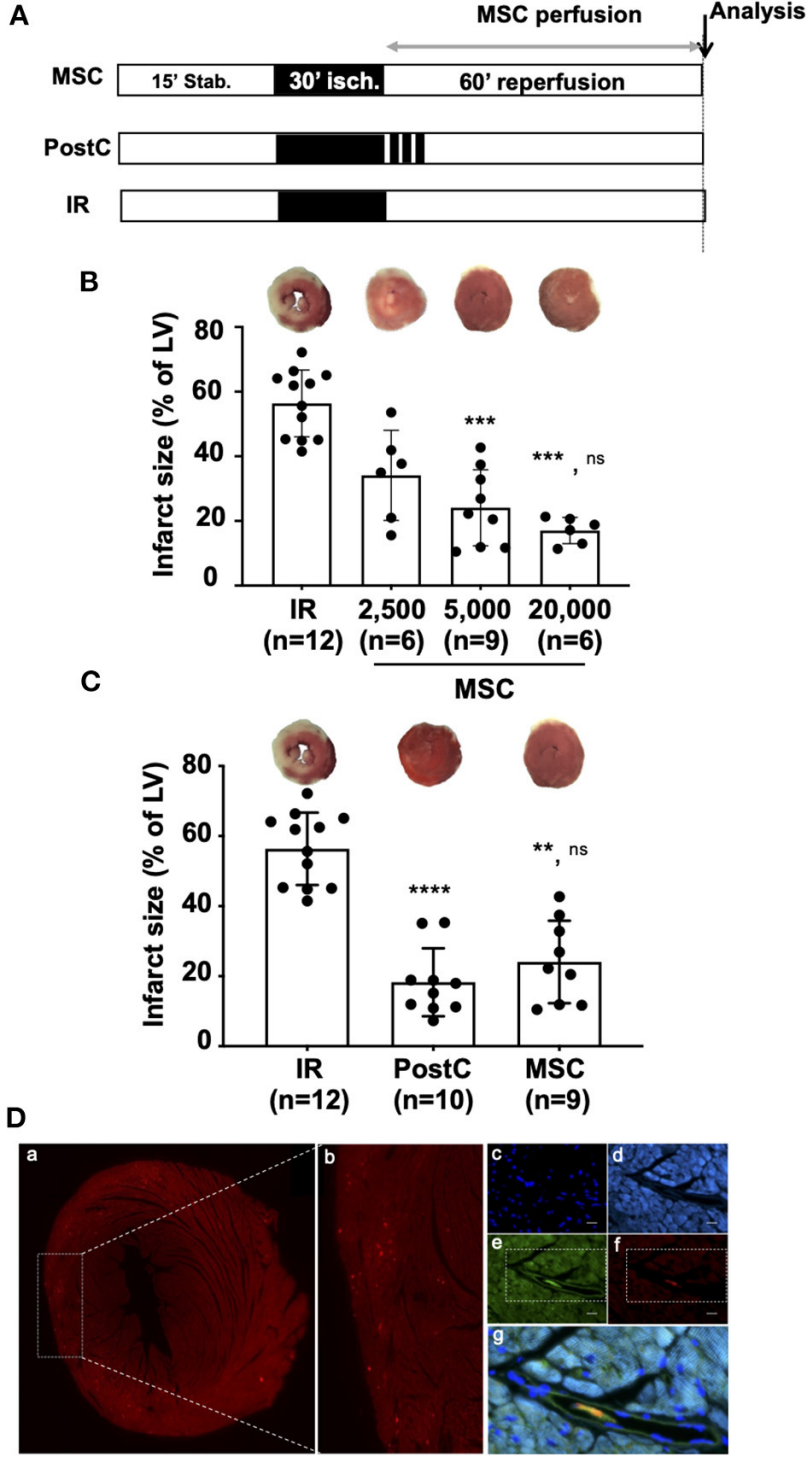
**(A)** Isolated hearts perfused *ex vivo* on the Langendorff system were submitted to perfusion protocol similar to that described in Figure 1A. In the MSC group, reperfusion was achieved with a solution of MSC cells prepared in a Tyrode buffer at various concentrations (2,500; 5,000; or 20,000 cells/mL). For the PostC group, a postconditioning stimulus comprising three cycles of 1 min ischemia-1 min reperfusion was applied at the onset of reperfusion. In the control condition (IR), hearts were reperfused with Tyrode solution alone (control condition). Histological analysis was performed at the end of the protocols for infarct size measurement and immunochemistry. **(B)** Scatter plots and bars (mean ± SD) were represented for infarct size (in % of LV) in IR (*n* = 12), MSC 2,500 cells/ml (*n* = 6), MSC 5,000 cells/ml (*n* = 9), and MSC 20,000 (*n* = 6). Representative pictures of TTC-stained LV slices were shown for each group. Statistical analysis was performed using Kruskal-Wallis with the Dunn's *post hoc* test for multiple comparison. Statistical significance is noted ^***^ for *p* = 0.0009 (MSC 5,000 vs. IR), ^***^ for *p* = 0.0003 (MSC 20,000 vs. IR) and ns for *p* > 0.99 (MSC 20,000 vs. MSC 5,000). **(C)** Scatter plots and bars (mean ± SD) were represented for infarct size (in % of LV) in IR (*n* = 12), PostC (*n* = 10), and MSC (5,000 cells/ml, *n* = 9). Representative pictures of TTC-stained LV slices were shown for each group. Statistical analysis was performed using Kruskal-Wallis with the Dunn's post hoc test for multiple comparison. Statistical significance is noted ^****^ for *p* > 0.0001 (PostC vs. IR), ^**^ for *p* = 0.0018 (MSC vs. IR) and ns for *p* > 0.99 (PostC vs. MSC). **(D)** Representative pictures of microscopic observations among 12 (data not shown) for an MSC-treated heart section **(a)** and **(b)** corresponding enlarged immunostaining images (Original magnification: x40 oil immersion) showing **(c)** cell nuclei (DAPI), **(d)** alpha-actinin, **(e)** microvessels (isolectin B4), **(f)** DI-I labeled MSC, and **(g)** merge allowing to show that MSC are located in the microvessels after 60 min of reperfusion (same time point of infarct size evaluation).

To reduce IR injury-related inflammation and thus infarct size, we used two well-known cardioprotective strategies, ischemic PostC and MSC-based therapy, to compare for the first time their effects in an *ex vivo* mouse model. Mesenchymal stem cells were administered during reperfusion in isolated hearts subjected to 30 min of global ischemia as described in the protocol shown in Figure 2A. Different concentrations of MSC in the perfusion solution were tested (Figure 2B). The dose-response curve was established using different concentrations of MSC and showed that 5,000 cells/mL (6.10^5^ cells/heart) and 20,000 cells/ml (24.10^5^ cells/heart) induced cardioprotective effects by decreasing infarct size (24.01% ± 11.8, *n* = 9 for MSC 5,000 vs. 56.4% ± 10.3, *n* = 12 for IR; *p* = 0.0009, and 17.0% ± 4.1; *n* = 6 for MSC 20,000 vs. 56.4% ± 10.3, *n* = 12; *p* = 0.0003) as opposed to 2,500 cells/ml (3.10^5^ cells/heart), which had no significant beneficial effect (34.1% ± 13.9, *n* = 6 for MSC 2,500 vs. 56.4 % ± 10.3, *n* = 12 for IR; *p* = 0.1571). The concentration of 5,000 cells/ml was chosen for all experiments in our study because it was the minimum concentration giving maximum cardioprotection in the *ex vivo* mouse model. Furthermore, we demonstrated that reperfusion with MSC at 5,000 cells/ml (6.10^5^ cells/heart) reduced infarct size to the same extent as ischemic PostC, taken as a positive control in our experiments (18.3 % ± 9.7, *n* = 10 for PostC vs. 24.1% ± 11.8, *n* = 9 for MSC 5,000; *p* > 0.99). Immunohistological analysis revealed that *ex vivo* perfused MSC labeled with CM-DiI were 100% co-localized with Isolectin B4-positive endothelial cells in coronary microvessels after 1 h of reperfusion (Figure 2D).

Altogether, these results demonstrate that 6.10^5^ MSC/heart provide a similar cardioprotection to that of PostC, considered as a positive control in our study.

### The Potent Cardioprotective Effect of Both Ischemic PostC and MSC Is Associated With a Decrease in Pro-inflammatory Cytokines in the Injured Myocardium

**Figure 3:**
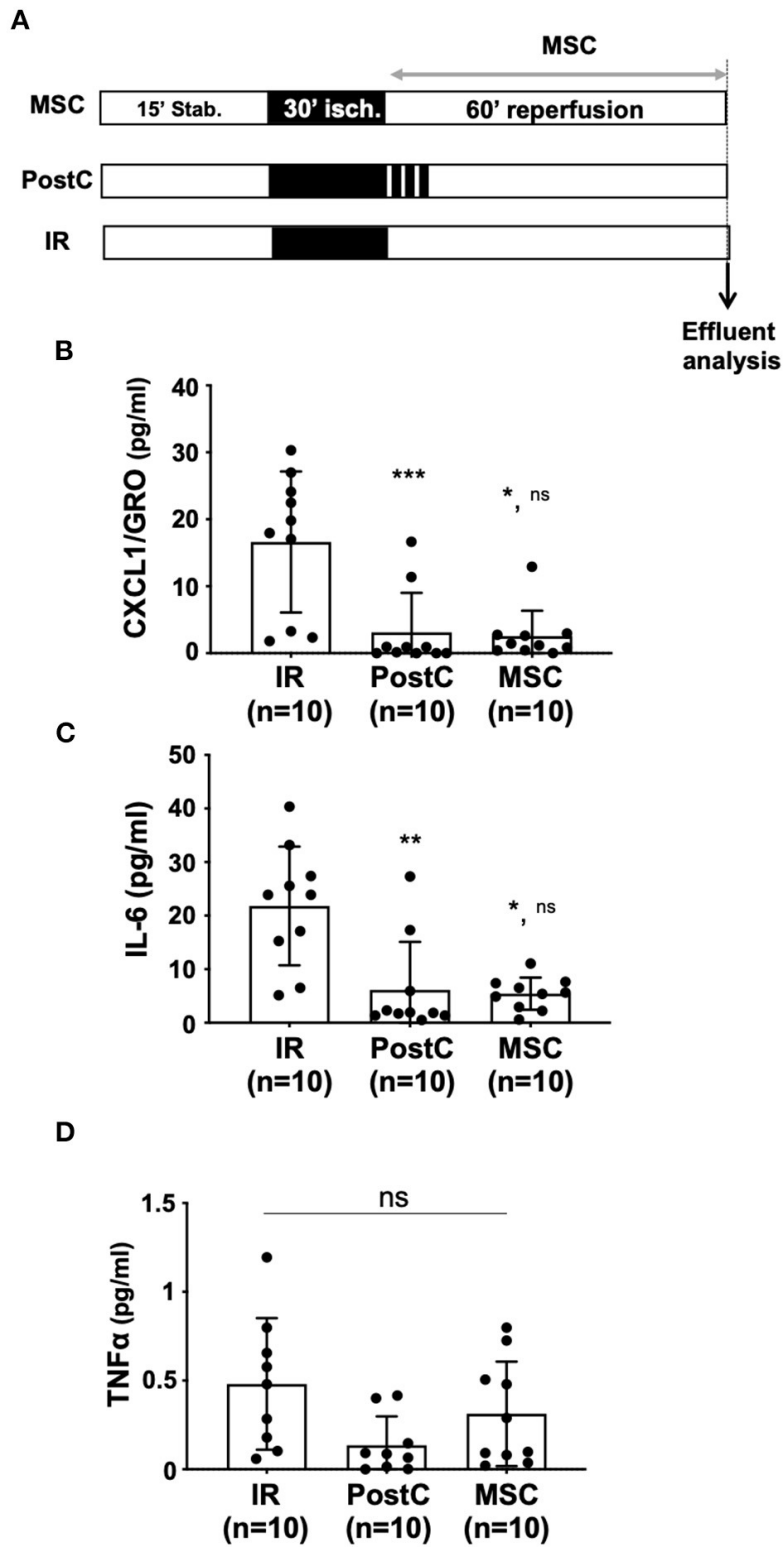
**(A)** Isolated hearts perfused *ex vivo* on the Langendorff system were submitted to the perfusion protocol similar to that described in Figure 1A. In the MSC group, reperfusion was achieved with a MSC Tyrode solution (5,000 cells/ml). For the PostC group, a postconditioning stimulus comprising three cycles of 1 min ischemia-1 min reperfusion was applied at the onset of reperfusion. In the control condition (IR), hearts were reperfused with Tyrode solution alone (control condition). Coronary effluents were collected at the end of the reperfusion phase to evaluate cytokine production after IR, PostC or MSC protocols. **(B–D)** Scatter plots with bars (mean ± SD) are presented for quantification of cytokines within coronary effluents collected after 60 min of reperfusion using the Meso Scale Discovery (MSD) V-Plex Plus Proinflammatory Panel 1 (mouse) kit. Statistical analysis was performed using the Kruskal-Wallis test followed by the Dunn's post test. **(B)** For CXCL1 (pg/mL), ^***^ was noted for *p* = 0.0006 (PostC vs. IR), ^**^ for *p* = 0.018 (MSC vs. IR) and ns for *p* > 0.999 (MSC vs. PostC). **(C)** For IL-6 (pg/ml), ^**^ was noted for *p* = 0.0021 (PostC vs. IR), ^*^ for *p* = 0.0287 (MSC vs. IR) and ns for *p* > 0.999 (MSC vs. PostC) and **(D)**: for TNFα (pg/ml), ns was noted for p = 0.059.

To determine whether the beneficial effect of PostC or MSC administration on IR injury was associated with the regulation of the immune response, we quantified pro-inflammatory cytokines in coronary effluents collected at 15, 30, and 60 min after the onset of reperfusion (Figure 3A). Among the 10 cytokines quantified by the MSD approach, CXCL1 (KC/GRO), IL-6, and TNFα, were detected with significant levels only at 60 min of reperfusion in the samples from the untreated IR hearts. IFNγ, IL-1β, IL-2, IL-4, IL-5, IL-10, and IL-12p70 were not detected. None of the 10 cytokines were detectable at 15 and 30 min post-reperfusion (data not shown). In addition, the levels of CXCL1 (KC/GRO) (Figure 3B) were significantly lower in coronary effluents from hearts treated by PostC or MSC (16.61 pg/ml ± 10.53, *n* = 10 for IR vs. 3.10 pg/ml ± 5.9, *n* = 10 for PostC; p^***^ = 0.0006, and vs. 2.56 pg/ml ± 3.79, *n* = 10 for MSC; p^*^ = 0.018). Similar results were obtained for IL-6 (21.83 pg/ml ± 11.08, *n* = 10 for IR vs. 6.16 pg/ml ± 8.95, *n* = 10; p^**^ = 0.021, and vs. 5.43 pg/ml ± 3.02, *n* = 10 for PostC; p^*^ = 0.029 for MSC; Figure 3C). For TNFα, there was a slight reduction of the effluent levels upon PostC treatment (without reaching significativity), which was not observed in the MSC group (*p*^ns^ > 0.99; Figure 3D).

This result indicates that the protective effects of both PostC and MSC were associated with a potent anti-inflammatory effect assessed by quantification of pro-inflammatory cytokines in coronary effluents.

### PPARβ/δ Is Involved in the Cardioprotective Effects Mediated by MSC Against IR Injury

**Figure 4:**
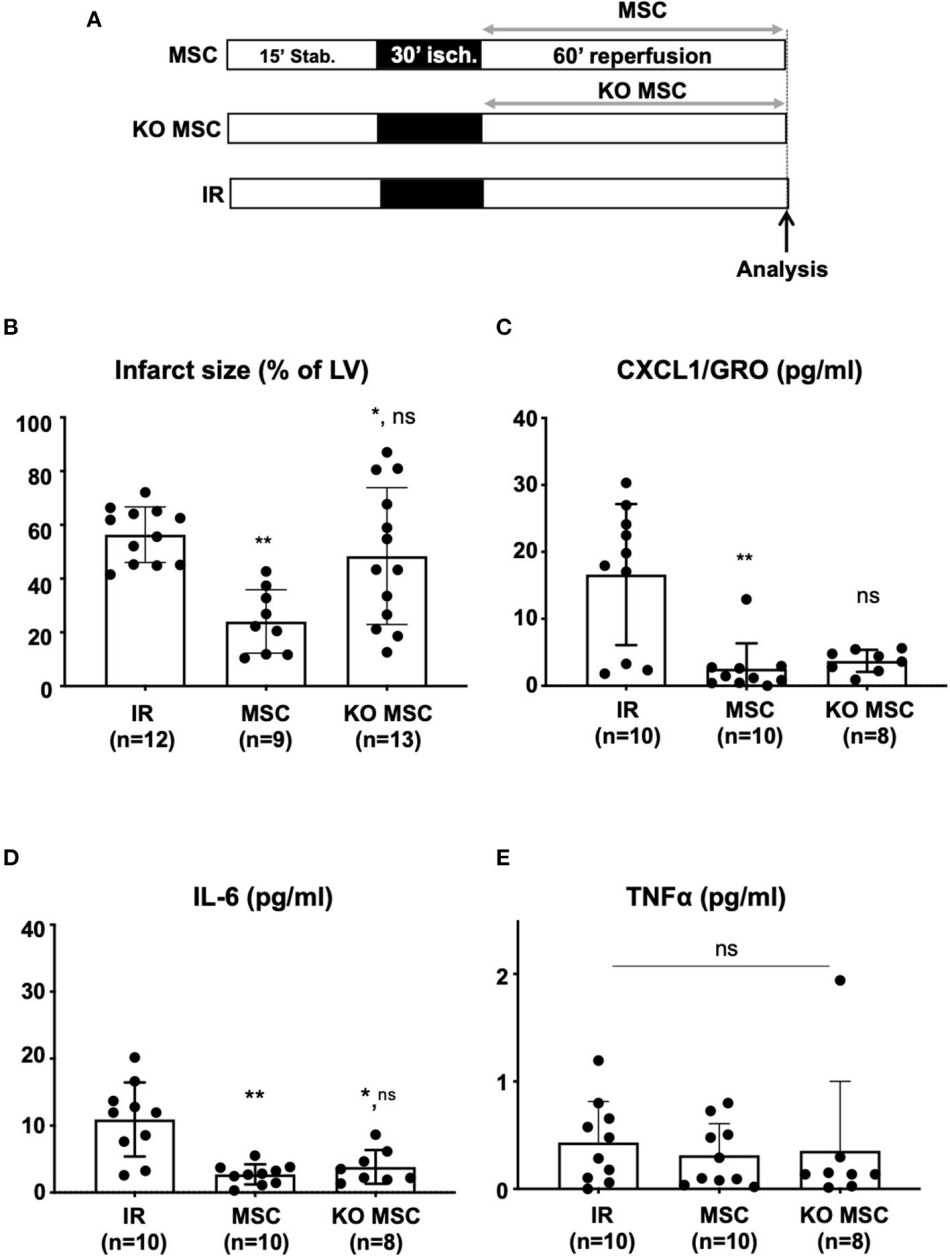
**(A)** Isolated hearts perfused *ex vivo* on the Langendorff system were submitted to perfusion protocol similar to that described in Figure 1A. Reperfusion was achieved with a Tyrode solution alone in the IR group or with a Tyrode solution prepared at a concentration of 5,000 cells/ml with MSC (MSC group) and with KO MSC (KO MSC group). At the end of the protocol, infarct size analysis was performed on the isolated heart **(B)** and cytokine production was analyzed **(C–E)** using the Meso Scale Discovery (MSD) V-Plex Plus Proinflammatory Panel 1 (mouse) kit. Statistical analysis was performed using the Kruskal-Wallis test with the Dunn's post test. **(B)** Scatter plots and bars (mean ± SD) were represented for infarct size (in % of LV) in IR (*n* = 12), MSC (5,000 cells/ml, *n* = 9), and KO MSC (5,000 cells/ml, *n* = 13). Statistical significance is noted ^**^ for *p* = 0.001 (MSC vs IR), ^*^ for *p* = 0.029 (KO MSC vs. IR), and ns for *p* = 0.075 (KO MSC vs. MSC). **(C–E)** Scatter plots with bars (mean ± SD) are presented for quantification of TNFα, CXCL1, and IL-6 within coronary effluents collected at 60 min after the onset of reperfusion from untreated hearts (IR), hearts treated with wild-type MSC (MSC) and MSC deficient for PPARβ/δ (KO MSC) using the Meso Scale Discovery kit. Statistical analysis was performed using the Kruskal-Wallis test. **(C)** For CXCL1 (pg/ml), ^**^ was noted for *p* = 0.0017 (MSC vs. IR), ns was noted for *p* = 0.34 (KO MSC vs. IR) and for *p* = 0.28 for (KO MSC vs. MSC); **(D)** For IL-6 (pg/ml), ^**^ was noted for *p* = 0.0021 (MSC vs. IR), ^*^ for *p* = 0.028 (KO MSC vs. IR), and ns for *p* > 0.999 (KO MSC vs. MSC); **(E)** for TNFα (pg/ml), ns was noted for *p* = 0.60.

Recently, we showed that PPARβ/δ is pivotal for the MSC immunoregulatory and therapeutic functions in an experimental model of arthritis (28). However, the role of PPARβ/δ on the cardioprotective activity of MSC and the relevance of PPARβ/δ to the anti-inflammatory properties of MSC in the inflamed myocardium have never been addressed. To determine whether PPARβ/δ is essential for the cardioprotective properties of MSC, we compared the effect of MSC isolated from PPARβ/δ^−/−^ deficient mice (KO MSC) and those obtained from their PPARβ/δ^+/+^ control littermates (MSC). Isolated hearts were perfused during reperfusion with solutions containing MSC at the optimal dose of 6.10^5^cells/heart (see protocol Figure 4A). Under these conditions, the drastic decrease in infarct size induced by MSC (24.1% ± 11.8, *n* = 9 for MSC vs. 56.4% ± 10.3, *n* = 12 for IR; p^**^ = 0.001) was abolished when KO MSC were infused into isolated hearts after the ischemic insult (48.4% ± 25.4, *n* = 13 for KO MSC vs. 56.4% ± 10.3, *n* = 12 for IR; *p*^ns^ = 0.75 and 48.4% ± 25.4, *n* = 13 for KO MSC vs. 24.1% ± 11.8, *n* = 9 for MSC; p^*^ = 0.029) (Figure 4B). A similar absence of cardioprotective effet was observed after the infusion of MSC pharmacologically inactivated for PPARβ/δ using GSK0660, a selective antagonist of PPARβ/δ (data not shown). To determine whether the loss of therapeutic effect of MSC in response to PPARβ/δ knockdown was associated with a loss of their ability to reduce inflammation in infarcted myocardium, we quantified pro-inflammatory cytokines within coronary effluents collected 60 min after the onset of reperfusion. Quantification of cytokines by MSD was performed in coronary effluents from hearts treated with either MSC or KO MSC and compared with those of untreated hearts. We demonstrated that PPARβ/δ knockdown in MSC did not alter their anti-inflammatory potential as revealed by the measured levels of CXCL1 (2.56 pg/ml ± 3.79, *n* = 10 for MSC vs. 3.73 pg/ml ± 1.65, *n* = 8 for KO MSC, *p*^ns^ = 0.28; Figure 4C) and IL-6 (5.43 pg/ml ± 3.02, *n* = 10 for MSC vs. 7.65 pg/ml ± 5.04, *n* = 8 for KO MSC, *p*^ns^ > 0.99; Figure 4D) in coronary effluents collected during reperfusion. There was no difference in the TNFα levels among groups (*p*^ns^ = 0.60; Figure 4E).

## Discussion

Our study evaluated, for the first time, the role of PPARβ/δ in MSC-induced cardioprotective effects in an *ex vivo* mouse model of myocardial IR injury. The rationale comes from data recently reported from our laboratory showing a pivotal role of these receptors for the immunoregulatory and therapeutic functions of MSC in an experimental model of arthritis (28). We first demonstrated that the *ex vivo* protocol of global ischemia (30 min) followed by 60 min-reperfusion used in that study induced a proinflammatory response assessed by an increase in the level of TNFα, IL-6, and CXCL1 in coronary effluents collected at 60 min of reperfusion. We then identified the optimal dose of MSC, 6.10^5^ cells/heart, to provide significant cardioprotection in the *ex vivo* mouse model with minimal cell concentration. Of note, at this selected dose of MSC, we observed similar cardioprotection to that of ischemic postconditioning (PostC) taken as a positive control in our study. Indeed, both treatments reduced the pro-inflammatory response of IR injury and decreased infarct size with the same efficacy. Moreover, this study revealed that the acute cardioprotective properties of MSC during the first hour of reperfusion are PPARβ/δ-dependent but not related to their anti-inflammatory effects on the release of CXCL1 and IL-6 in coronary effluents.

Numerous studies have demonstrated in isolated perfused hearts (*ex vivo*) the cardioprotective effect of PostC or various pharmacologic drugs applied at the onset of reperfusion. Considering most *in vivo* studies, the protocol is quite similar since PostC or protective drugs are applied at the onset of reperfusion and infarct size measured often at 1 h reperfusion, an effect maintained at 24 h or at several months of reperfusion (39). This means that major local events occur very early during reperfusion. These rapid local events, which are triggered during reperfusion, include inflammatory processes and/or paracrine effects of secreted molecules such as cytokines by endogenous or exogenous cells. The *ex vivo* model is well-suited to identify these pathways, as short-term protection is critical for long term protection.

Despite its benefical effects in AMI patients, reperfusion therapy induces local and systemic inflammation, termed sterile inflammation, which will enhance the initial inflammatory response triggered to clear necrotic cells after AMI and to repair the infarcted myocardium. To develop and evaluate innovative therapeutic approaches, we designed an *ex vivo* model that provides strong IR injury assessed by 56% infarct size (expressed as percentage of the LV) and the release of pro-inflammatory cytokines at significant levels in coronary effluents, as reported *in vivo* in patients with acute coronary syndrome (40). This inflammatory response is a potential therapeutic target to improve the post-AMI clinical state because it plays a crucial role in determining infarct size and subsequent left ventricular remodeling. To protect the myocardium, many therapeutic strategies targeting indirectly or directly the proinflammatory response after AMI have been tested. In this context, approaches to decrease the levels of inflammatory cytokines and chemokines in animal models have been developed and used with promising results regarding the control of inflammation (2). However, most clinical trials based on the use of broad-spectrum immunosuppressive drugs such as corticosteroids or immunosuppressants in AMI patients have shown no benefit on infarct size or clinical outcomes (2). Since rapid activation of this inflammatory response is a consequence of an inappropriate activation of the innate immune system triggered by DAMPs release, targeting key components of the innate immune system appeared as a promising approach for limiting IR injury (41). Thus, more refined strategies have been proposed instead of fully suppressing the immune system. In this context, promising results have been obtained although they have been mitigated by negative results as reported for inhibitors of IL-1 (42, 43). Targeting inflammation in AMI patients is quite challenging because innate immunity has been reported to contribute to myocardial repair and remodeling (44). This makes it difficult to determine the right dose and timing of administration of therapeutic agents to avoid compromising innate immunity-induced cardiac repair. Mesenchymal stem cells-based therapy in this context appears as an interesting strategy in creating local inflammation permissive to regeneration.

Ischemic postconditioning, considered the gold standard of cardioprotection against IR injury, has been reported to activate a myriad of intracellular cascades leading to inhibition of regulated cell death and also an anti-inflammatory response to induce a strong cardioprotective effect at 1 h of reperfusion (9). In the *ex vivo* model of IR injury used in the present study, the PostC cardioprotective strategy reduced both infarct size by 75.1% (PostC vs. IR, p^*^) and IL-6 and CXCL1 levels by 71.8 and 91.4% compared with IR, respectively, in coronary effluents from isolated hearts. Taken together, these results suggest that regulation of the inflammatory response is associated with cardioprotection against IR injury mediated by PostC.

Therefore, global therapies such as PostC, have emerged as promising approaches to treat multifaceted ischemic heart disease and restore cardiac function. Indeed, considering the pleiotropic effects of MSC that include their immunoregulatory, antifibrotic, and anti-apoptotic capabilities, MSC-based therapy could counteract the three main pathogenic axes of AMI and thus have been considered a breakthrough in this incurable disease with unmet medical needs. Safety and efficacy were evaluated by assessment of adverse events and the improvement of the left ventricular ejection fraction (LVEF) and mortality rate, respectively. Although the results obtained were marked by significant heterogeneity, MSC injection did not appear to be associated with acute adverse events but induced an improvement in LVEF in patients. No significant differences in mortality were reported. Other outcomes of interest were rarely studied, which limits the conclusions. In our study, MSC exerted a cardioprotective effect similar to that provided by PostC, considered a positive control in our study. Indeed, infarct size was decreased by 69.3% using MSC-based cell therapy (PostC vs. MSC, *p* = ns). In addition, MSC treatment (6.10^5^ cells/heart) had a strong anti-inflammatory effect, as assessed by MSD quantification of IL-6 and CXCL1, which were decreased by 75.1 and 84.6%, respectively.

In this context, we tested the therapeutic role of MSC knockdown for PPARβ/δ previously described by our laboratory to exhibit an enhanced ability to inhibit both T-cell proliferation, *in vitro*, and arthritic development and progression in CIA *in vivo* compared with naive MSC (28). When primed with TNFα and IFNγ, MSC deficient for PPARβ/δ express increased levels of mediators of MSC immunosuppression, including VCAM-1, ICAM-1, and nitric oxide (NO), compared with their wild-type counterparts (28).

In the present study, we observed that knockdown of PPARβ/δ in MSC did not alter their anti-inflammatory properties when injected into the infarcted myocardium. Indeed, both wild-type and PPARβ/δ KO MSC significantly decreased pro-inflammatory cytokine levels within coronary effluents after 60 min of reperfusion after an ischemic insult. Therefore, the loss of MSC therapeutic effect in the myocardium subjected to IR injury reported here for PPARβ/δ KO MSC could be attributed to an impairment of other functions of MSC pivotal for their beneficial effect in AMI or to a reduction of their survival in perfused hearts. We have recently shown that PPARβ/δ modulation impacts on MSC metabolism (45). Indeed, inactivation of PPARβ/δ promoted the metabolic switch of MSC from oxidative phosphorylation to glycolysis (45). This is in agreement with the role of PPARβ/δ previously described in energy-demanding cells and tissues in which PPARβ/δ promotes fatty acid oxidation that leads to increased ATP production contributing, not only to cell survival but also to cell protection and maintenance of its function (46).

Therefore, it is tempting to anticipate that PPARβ/δ deficiency in MSC could affect their survival rate and reduce their engraftment once injected *in vivo* since PPARβ/δ has been described to promote survival of several cell types, including cancer cells and cardiomyocytes (47, 48) [for review see (49)]. In endothelial cells, anti-apoptotic role of PPARβ/δ has been reported and the underlying mechanisms were related to endothelial 14-3-3 upregulation (50, 51). Similarly, in the cardiomyoblast cell line H9c2, activation of PPARβ/δ by the selective agonist, GW501516, was described to protect the heart from H_2_O_2_-induced cell death (52). PPARβ/δ is highly expressed by MSC (28) but its role on MSC anti-apoptotic and cardioprotective properties has never been investigated. Moreover, further studies are required to identify key mediators regulated by PPARβ/δ and involved in the acute cardioprotective effect of MSC.

In conclusion, our study shows for the first time that PPARβ/δ plays a key role in the acute local cardioprotective effect of MSC against myocardial IR injury *ex vivo*. Moreover, PPARβ/δ knockdown does not affect the anti-inflammatory properties of MSC assessed in isolated hearts after 1 h of reperfusion. Altogether, these results highlithing the crucial role of PPARβ/δ in MSC cardioprotective properties pave the way toward the development of novel strategies for MSC-based therapy for AMI patients. Further *in vivo* studies will be required to evaluate the contribution of the peripheral immune system to the inflammatory response of IR injury and the subsequent cardiac remodeling.

## Study Limitation

This study had some limitations because the coronary flow was not assessed in our *ex vivo* experiments performed on a conventional Langendorff system (glass coils and tubes) in contrast to previous studies in our laboratory performed with a fully integrated system (discarded here because cells adhered to the long polyethylene tubes) (53, 54). This technical limitation could introduce biases in the quantification of cytokines in the perfusates because the concentration of circulating molecules depends on the coronary flow.

To interpret results on Figure 1C, we can refer to data from the literature obtained with the same *ex vivo* model of 30-min global ischemia followed by 60 min of reperfusion showing that coronary flow at 60 min of reperfusion is about 80% of baseline (55). In this context, the decrease in coronary flow at 60 min of reperfusion could contribute to the increase in the measured levels of cytokines in the perfusates compared with basal conditions. Our interpretation was toward an increased release of proinflammatory cytokines in our IR model, consistent with the widely reported pro-inflammatory effects of IR injury (2) even *in vivo* in patients with acute coronary syndrome (40). Considering the results presented in Figure 3C, we observed decreased amounts of cytokines (CXCL1 and IL-6) in the PostC condition vs. IR, whereas Maruyama et al. have reported similar values of coronary flow values at 60 min of reperfusion in both IR and PostC conditions (55). On the basis of these results, we can therefore suggest that the decrease in cytokine levels at IR60 observed after a PostC (see Figure 3C) may result mainly from a decrease in the release of cytokines vs. IR, in accordance with the anti-inflammatory effect of PostC described *in vivo* (10, 56). However, we cannot exclude an improvement in the coronary flow upon PostC, as described for many other cardioprotective strategies evaluated in our laboratory (53, 54) or others (57), which could also contribute to the reduction in measured amounts of cytokines.

Considering MSC effects (Figures 3, 4), the authors are confident in their interpretation of the results showing a decrease in cytokine release vs. IR, consistent with the anti-inflammatory effect of MSC widely described in the literature (14, 15). Furthermore, these results are corroborated by those showing reduced levels of IL-6 in the serum of pigs subjected to myocardial IR injury *in vivo* (58) and, more importantly, by other data showing decreased protein production and gene expression of inflammatory cytokines (including IL-6) that are not dependent on the coronary flow (20). In addition, it can be assumed that the coronary flow might be decreased when MSC are administered during reperfusion because of their relatively large size in small-diameter coronary vessels, as suggested by *in vivo* data showing microvascular obstruction after intracoronary injection of MSC (59–61), especially with high doses of MSC (44.10^6^ cells) leading to an increase in intravascular resistance, a subsequent decrease in coronary flow, and also to microinfarction (62, 63). However, this deleterious effect does not seem to occur here in our experiments because (i) the mean values of cytokine levels assessed in the MSC group were close to those obtained for PostC (Figures 3B,C), (ii) the dose of MSC was relatively low (6.10^5^ cells/heart), and (iii) a drastic decrease in infarct size was observed upon MSC treatment (Figures 2B,C, 4B).

Overall, despite the absence of coronary flow assessment, the results on decreased cytokine concentration presented here seem to be related to the anti-inflammatory effects of PostC and MSC treatment against IR injury.

## Data Availability Statement

The original contributions presented in the study are included in the article, further inquiries can be directed to the corresponding author/s.

## Ethics Statement

Studies involving animals were reviewed and approved by the Institute's SBEA (Structure Bien-être Animal) committee in accordance with the European directive 2010/63/EU and the French Ministerial Order of February 01, 2013.

## Author Contributions

FD and SB-L designed the all project and the experiments. CJ, CP, JN, and AV contributed to design the experimental protocols and interpret the data for the work. CS, CB, GT, RC, PL-C, and NN performed the experiments and analyzed the results. FD and SB-L wrote the manuscript with the input of CJ, JN, AV, SK, and CP. All authors revised and gave final approval of the manuscript.

## Funding

This work was supported by Inserm, the University of Montpellier (FD, SB-L, and CJ) and by the CNRS (SB-L). We thank the Fonds Marion et Elisabeth Brancher for the financial support of this project (CS) and also the PHC program of Campus France (project number 33858WM; SK and SB-L) as well as the Agence Nationale de Recherche for the LabEx ICST ANR (ANR-11-LABX-0015; SB-L, JN, AV, CB, and CP) and for the PPAROA ANR (ANR-18-CE18-0010-02; FD, CJ, and RC) grants, SATT AxLR n°19/0150 contract, the ECOS-Sud (action ECOS n°C18S03) and La Fondation Arthritis. We also thank the University of Naresuan for the staff development travel grant (NN).

## Conflict of Interest

The authors declare that the research was conducted in the absence of any commercial or financial relationships that could be construed as a potential conflict of interest.

## Publisher's Note

All claims expressed in this article are solely those of the authors and do not necessarily represent those of their affiliated organizations, or those of the publisher, the editors and the reviewers. Any product that may be evaluated in this article, or claim that may be made by its manufacturer, is not guaranteed or endorsed by the publisher.
